# The Desire of Parenthood: Intuitive Co-parental Behaviors and Quality of Couple Relationship among Italian and Belgian Same-Sex and Opposite-Sex Couples

**DOI:** 10.3389/fpsyg.2017.00110

**Published:** 2017-02-14

**Authors:** Marina Miscioscia, Adelaide Blavier, Paolo R. Pagone, Alessandra Simonelli

**Affiliations:** ^1^Department of Psychology and Clinics of Human Systems, University of LiègeLiège, Belgium; ^2^Department of Developmental and Social Psychology, University of PadovaPadova, Italy

**Keywords:** parenting desires/intentions, same-sex couples, co-parenting, dyadic adjustment, Prenatal Lausanne Trilogue Play

## Abstract

Studies that focused on family issues have allowed a great understanding of the aspects related to its subsystems, such as parenting desire and its expectations, couples’ satisfaction and quality of child’s outcomes. All these aspects are greatly interconnected and contribute to the creation of specific family dynamics, such as the quality of family interactions. The present study focuses on intuitive co-parental behaviors and the quality of couple relationship observed during the decision process (intention and desire) to be (or become) parents. Our first goal was to explore these aspects in a cross-national sample made of Italian and Belgian heterosexual, lesbian and gay couples. We then aimed to evaluate if the degree of internalized homophobia affects co-parental alliance. The quality of couple relationship and co-parental behaviors have been evaluated through the recruitment of a group of 115 stable heterosexual, gay and lesbian couples (230 individuals, 20–50 years of age) without children, who wanted to become parents. We used the Prenatal Lausanne Trilogue Play to evaluate the Co-parental Alliance; the couple’s satisfaction was assessed with the Dyadic Adjustment Scale and the Internalized Homophobia with the MISS-LG. In line with the existent literature, the analysis did not find any difference between lesbian, gay and heterosexual couples in terms of co-parental alliance. High levels of couple adjustment lead to better parental performances among both Italian and Belgian couples. The results suggest also that sexual stigma differs from one country to another, and it has an impact on the capability of managing co-parenting. Clinical implications should be verified in further longitudinal studies in order to observe the impact on the inter-generational transmission of psychopathology.

## Introduction

Over the past few years, we noticed an increase on LGBT (Lesbian, Gay, Bisexual, and Transsexual) family issues research, with a focus on family internal dynamics, in comparison with the first research field, which concentrated mostly on children’s outcomes (Patterson, 2005; Tasker, 2005; Peplau and Fingerhut, 2007), co-parental behaviors and couple’s adjustment (Farr and Patterson, 2013). These studies and reviews showed how lesbians and gay men could be parents as well as heterosexuals (Falk, 1989; Victor and Fish, 1995; Patterson and Chan, 1996; Brewaeys and Van Hall, 1997; Parks, 1998; Tasker, 1999; Patterson, 2000; Stacey and Biblarz, 2001; Anderssen et al., 2002; Perrin, 2002) and consequently, how motherhood and fatherhood do not relate to sexual orientation. Farr and Patterson (2013) examined differences between lesbian, gay and heterosexual couples studying correlations between self-reported division of the daily tasks, adopted children adjustment and co-parenting evaluation through an observational technique during a play session. All along the role-play, the authors assessed supportive (warmth, enjoyment, eyes contact) and undermining (to talk over the partner, to suggest different toys to the child) behaviors (McHale et al., 2001). Results have detected differences between the three groups in terms of labor division and co-parenting. Lesbian and gay parents were more likely than heterosexual parents to report sharing child-care. In addition, lesbian couples showed the most supportive interactions while gay couples showed the least. Heterosexual couples were intermediate between lesbian and gay couples in supportive behaviors. Despite those results, no differences have been found in terms of child adjustment between the three groups of families.

At the same time, several longitudinal studies, which were conducted with heterosexual-headed families, showed that early family interactions have a significant influence on a child’s emotional and cognitive development, in particular during the first years of the child’s life (Carneiro et al., 2006). Studies on the development of families, which include observations of parent-infant interactive behaviors, revealed that mother-father-infant interactions during infancy are predictive of emotional and cognitive outcomes in the child (Favez et al., 2012), especially for the development of the theory of mind assessed at age 5 (Frascarolo et al., 2008).

Yet, the interest shown for LGBT families’ issues concerning the quality of family interactions is quite recent. D’Amore et al. (2013) investigated differences between both healthy lesbian and heterosexual couples and a group formed by heterosexual couples with depressed mothers, using the Lausanne Trilogue Play paradigm (LTP; Fivaz-Depeursinge and Corboz-Warnery, 1999). The clinical approach detected no differences between healthy groups, independently from the sexual orientation, but rather between the two healthy groups and the group that included depressed mothers, explaining how much the maternal psychopathology could affect co-parental behaviors. In order to better understand on which levels it is necessary to intervene to encourage the healthy development of the family, future research is necessary to investigate which protective and risk factors are in common to all families, and which ones are specific of different families’ configuration.

### The Desire of Parenthood

Changes that accompany the process to parenthood are so deep; their impacts vary over time, from couple to couple, and from individual to individual (Delmore-Ko et al., 2000). Sommer et al. (1993, p. 389) found that individuals who were more “cognitively ready” to become parents had lower levels of parenting stress and were more prone to adapt in their parenting style. Researchers demonstrated the considerable impact that the birth of a first child has on many aspects of family life, and the importance of couples’ expectations in predicting how they will adjust to these changes. The following factors are protective ones for the health of both the family and the child: a stable relationship, the support of the family of origin and an appropriate development of the individual (the future parent) and the couple desire.

The relationship quality has been shown to influence women’s feelings about childbearing. Fischer et al. (1999) found that women were more likely to want a pregnancy if they expressed positive feelings about their partner. The expectation about the duration of the relationship is another measure related to the stability of the relationship, and this measure has been found to influence the desire of having a baby (Wilson and Koo, 2006). Some studies suggested that women who do not have children with their current partner, even if they already have ones from previous romantic relationships, would want to have at least one child with him in order to achieve their concept of “family” (Thornton, 1978; Hoffman and Manis, 1979) or to strengthen the relationship (Westoff, 1977).

Riskind and Patterson (2010) published national representative data about parenting intentions and desires in a sample of American childless lesbian, gay and heterosexual people, and they found that 37% of childless lesbian participants expressed a desire for children, compared with 68% of heterosexuals women. On the contrary, 54% of childless gay men expressed a desire for children compared to 67% of heterosexual men. Despite this difference between gay men and lesbians, gay men who reported the desire to become parents were less likely to also express the intention of becoming parents than heterosexual men. While lesbians who expressed the desire of becoming parent expressed the intention of it as well (Riskind and Patterson, 2010).

D’Augelli et al. (2007), in an American sample of urban lesbian and gay youths, found strong expectations of parenthood among lesbian (91%) and gay youths (86%). Similarly, Gates et al. (2007) discovered that childless gay men were less likely to express a desire for children (52–67%), compared with childless heterosexual men.

Previous research showed that among gay- and lesbian-headed families the asymmetry between gender role (masculine and feminine) and parental role (paternal and maternal) requests a continuous redefinition of tasks, in particular concerning child-rearing, which does not reflect the parents’ biological genders (Coltrane, 2000). Lesbian and gay couples, for example, often report to divide child-care more equally than heterosexual couples, who, on the contrary, report role specialization (Goldberg, 2010). Despite this difference, same-sex couples and opposite-sex couples declare the same degree of satisfaction about their relationship (Sullivan, 1996; Tasker and Golombok, 1998; Johnson and O’Connor, 2002; Bos et al., 2004; Patterson, 2005). Patterson (1995a,b) revealed differences among lesbian couples with a biological mother and a non-biological mother. The author showed that co-mothers were not different in the quantity of involvement, but in the quality: biological mothers spent more time in the real child-caregiving, whereas non-biological mothers did it in activities and playtime. Bos et al. (2007) showed that biological mothers use more authority than non-biological mothers, and non-biological mothers happen to be less rigid and more involved than heterosexual fathers. Despite these qualitative differences, the involvement in caregiving is distributed more equally among lesbians (Chan et al., 1998; Ciano-Boyce and Shelley-Sireci, 2002). Other studies regarding lesbian motherhood showed that mother-child interaction patterns are similar with opposite-sex parents’ ones (Vanfraussen et al., 2003) or even better (Brewaeys and Van Hall, 1997).

In our opinion, the observation of the transition to parenthood, which shows the way a family develops, could be useful to extend the comprehension about protective and risk factors. The assumption is that family is an already created concept in couples’ minds even before a real family is formed.

### Co-parental Behaviors and Quality of Couple Relationship

Studies about family system have stressed a distinction between “marital couple” and “co-parental couple” (Cowan and McHale, 1996; Katz and Gottman, 1996; McHale and Fivaz-Depeursinge, 1999; McHale, 2007; Simonelli et al., 2012). The first one refers to everything regarding the relationship between two adults partners mutually bound (Simonelli et al., 2012). Co-parenting describes the synchronization between adults in their parental roles (Minuchin, 1974) and it refers to their capacity to share work and responsibilities toward child caregiving. Co-parenting refers to the competence of mutual support and coordination between adults who are responsible for childcare and child rearing (McHale, 2007).

Researchers and theorists developed co-parenting notions, in studying the cooperation between parents-to-be by the use of observational techniques administered during the pregnancy (Carneiro et al., 2006), when both behavioral and mental aspects of parenting are triggered (Simonelli et al., 2012). According to Corboz-Warnery and Fivaz-Depeursinge (2001), the cooperation between parents appears during the pregnancy through intuitive behaviors (Papoušek and Papoušek, 1987), which anticipate the first encounter with the infant. Longitudinal studies that used the prenatal Lausanne Trilogue Play (prenatal LTP; Carneiro et al., 2006) showed that the interactions in the pregnancy period, with a fake baby (doll), were predictive of the future interactions with the real baby, after the childbirth (Favez et al., 2006). Marital and co-parental relationships are both important for the child’s outcomes (Belsky, 1984), but co-parenting has been found to be more strongly related to the child’s adjustment than other factors of couple relationship (Farr and Patterson, 2013). In fact, dysfunctional difficulties, at a co-parental level, could adversely affect the child’s outcomes (Minuchin and Minuchin, 1987) and promote the development of negative cognitive, social and emotional factors (McHale, 2007; Gatta et al., 2016a,b). Co-parenting could be one of the most important and influent mediator factors for functional family patterns, which ensures a good child’s development.

### Aims

According to the literature references presented above, the aim was to investigate co-parental alliance in order to (i) observe similarities or differences concerning co-parenting among stable gay, lesbian and heterosexual couples without children; (ii) evaluate, among LG couples, the correlation between co-parental dimension, dyadic adjustment, internalized homophobia and social support.

We suppose that there are no differences among the three types of couples when it comes to the quality of co-parental alliance. On the contrary, we hypothesize that couples with “better quality” of relationship and “high social support” show “better quality” in co-parental alliance, and that the internalized homophobia affects co-parental skills among LG couples.

This research also aimed to explore if the availability of legal marriage within a particular country influences the ways in which LG couples manage their transitions to parenthood and co-parental alliances. We also evaluate in what extent the degree of internalized homophobia affects co-parental alliance. We hypothesize that high levels of internalized homophobia lead to low scores of co-parental alliance.

We also assume that participants from two different countries, with different legislations and civil rights for LGBT people, show a different degree of internalized homophobia. For this purpose, this paper focuses on two groups of gay and lesbian couples living in two different European countries: Italy and Belgium.

### Civil Rights for LGBT People

The choice to involve subjects from Italy and Belgium is motivated by the legal differences existing at the time of the recruitment (October 2014–December 2015) in terms of LG population’s civil rights. Belgium represents one of the leading countries in recognizing and protecting civil rights among LGBT people. The “Europe Annual Review of the Human Rights Situation of LGBTI People in Europe” (2014), published by the ILGA (International Lesbian and Gay Association [ILGA] Europe, 2014), sees Belgium in second position over 49 European countries for LGBTI (Lesbians, Gay, Bisexuals, Transsexuals, and Intersexuals) issues, whereas Italy ranks 32nd. Since 2003, Belgium legally recognizes same-sex couples by civil marriage, and, since 2006, it allows the adoption and the access to medically assisted procreation. On the contrary, Italian legislation is still incomplete in terms of legal protection of gay and lesbian community’s rights. Indeed, during the recruitment, no legislation regarding same-sex civil unions existed in Italy, and such status was not even legally recognized.

## Materials and Methods

### Participants

One hundred and fifteen unmarried stable couples were recruited in Italy and Belgium (128 volunteers from Belgium with mean age = 24.3 years, *SD* = 3.7; 102 volunteers from Italy with mean age = 29.2 years, *SD* = 6.4). On **Table 1** it can be noticed that groups are different in terms of age and duration of relationship. Observing the groups average, Italian participants are older than the Belgian ones and they have been in their relationships longer.

**Table 1:** Mean and standard deviation of Age (years), duration of the relationship (in months) and coming-out age (years) of each group.

	Gay men (*N* = 68)				Lesbians (*N* = 62)				Heterosexuals (*N* = 100)			
			
	Italians *N* = 34	Belgians *N* = 34			Italians *N* = 38	Belgians *N* = 24			Italians *N* = 56	Belgians *N* = 44		
	*M(SD)*	*M(SD)*	*F*	*p*	*M(SD)*	*M(SD)*	*F*	*p*	*M(SD)*	*M(SD)*	*F*	*p*
Age	33.2*(7)*	25*(4.1)*	10.25	0.002^∗^	28.8*(5.6)*	24.2*(3.9)*	5.4	0.023^∗^	26.4*(4.6)*	23.9*(3.2)*	5.45	0.022^∗^
C.O.	21.4*(4.7)*	17.7*(3.9)*	0.842	0.363	21.4*(4.5)*	18.1*(3.6)*	2.2	0.144				
D.R.	62.1*(50.7)*	30.3*(22)*	33.02	0.000^∗∗^	40*(25.2)*	30.1*(13.6)*	16.7	0.000^∗∗^	50.7*(29.3)*	38.4*(21)*	5.85	0.017^∗^


*C.O., coming-out age; D.R., duration of the relationship.*

Inclusion criteria were: (i) being in a stable relationship for 1 year at least to be sure that the adult attachment was established, (ii) never been married, (iii) never had children [because we wanted to evaluate the relationship with the imaginary child (Lebovici, 1988) and not with a real baby], (iv) be supportive to the idea of becoming parents in the future.

Participants were recruited^1^ in northern Italy and Liège county in Belgium through web-posted advertisements, which informed that research was about “couples and parenting” without other explanations, in order to not induce biased answers. Thereupon, the sample is not randomized, but it could be defined as a “convenience” sample.

Each participant agreed to the informed consent and they gave their e-mails to allow follow-up contacts. After having submitted the answers, a researcher sent an e-mail to invite them in a specific laboratory^2^ for performing LTP and completing questionnaires.

### Materials

*Prenatal Lausanne Trilogue Play.* Prenatal LTP (Carneiro et al., 2006) is a peculiar LTP (Fivaz-Depeursinge and Corboz-Warnery, 1999) version. Authors created this observational tool to evaluate representations of the child-to-be during the pregnancy, in particular through the seventh month. This is the reason why none of the couples picked for this study was pregnant. Parents-to-be are invited to sit by a facilitator in a triangular configuration, with a basket as a vertex. A “neutral” doll, with the typical size and shape of a newborn, represents the baby. The face has features and traits of a Caucasian baby, “neutral” in relation to sex or particular eye, skin, and hair color. Such “neutrality” should help parents-to-be to role-play the situation. A camera records the entire procedure, standing in front of the parents, it slightly varies from the original LTP during which there is another camera recording the infant’s facial expression. The facilitator asks the parents to imagine the moment when the three of them would encounter for the first time after delivery. He/she explains that the task has four parts: (a) One of them would play with the baby doll, (b) then the other, (c) then the parents would play together with the baby, and finally, (d) they would let the infant “sleep” and then talk together about the experience that they just had. The exercise lasts about 5 min.

Prenatal co-parenting was assessed in the prenatal LTP situation using five scales on a Likert Scale ranging from 0 to 5. Carneiro et al. (2006) specifically elaborated the first three scales to analyze the prenatal LTP. The five scales are: (1) *Co-Parent Playfulness*, which assesses the capacity of the couple to co-construct playful games. The evaluation also concerns the ability of the couple to understand that the situation is a simulation and not the reality. (2) *Structure of the Play*, assesses the couple’s capacity to structure the four play parts. In this scale, two dimensions are considered: the differentiation of the play into four discrete segments and the duration of the entire play sequence as well as of each segment. (3) *Intuitive Parenting Behaviors*, assesses the parents’ use of intuitive parenting behaviors. Six behaviors that relate to literature (Papoušek and Papoušek, 1987) are coded: holding and “en face” orientation, dialog distance, baby talk and/or smiles at baby, cuddling and/or rocking, exploration of baby’s body, and preoccupation with the baby’s well-being. These intuitive parenting behaviors are assessed as present or absent for each parent. These individual results are mixed together into a global score for the couple. (4) *Couple Cooperation Scale*, assesses the degree of active cooperation between the parents during the play, at a behavioral level. And finally, (5) Family Warmth, captures the affection and mood shared by the partners during the play; namely, whether they manifest affection and tenderness as a couple and toward their “baby.”

Scores of the five scales are added to obtain a global score between 5 and 25. The higher is the score, the more the prenatal alliance is considered to be functional.

*Measures of Internalized Sexual Stigma for Lesbians and Gay Men. MISS-LG* (Lingiardi et al., 2012) is a doubled version scale, one for each gender, formed by 17 items on Likert Scale with five points (from “I disagree” to “I agree”). It quantifies internalized stigma degree, defined as the feelings expressed by gay men and lesbians toward homosexuality at large and toward themselves (ibidem). Two versions (L and G) are the same in 11 items, but differ in six that reflect gender differences. Scoring gives back four scores^3^ regarding three dimensions of the internalized sexual stigma and a total score that results from the average of all items. The higher is the total score, the more is the Internalized Sexual Stigma degree.

*Dyadic Adjustment Scale* (DAS; Spanier, 1976; Italian version translated and validated by Gentili et al., 2002; French version validated by Vandeleur et al., 2003). The 32 items of the scale assess several aspects of the couple’s life, such as the frequency and intensity of disagreements and/or agreements on the marital emotions, actions, and activities. The total of the answers display a score between 0 and 151: the higher the score is, the higher the couple is satisfied with its relationship. An average score is computed for every couple.

*The Interpersonal Support Evaluation List* (ISEL; Cohen and Hoberman, 1983; Italian version translated and validated by Moretti et al., 2012) evaluates how people behave when they need help during negative events. ISEL consists of a list of 40 statements concerning the perceived availability of potential social resources. The items are counter-balanced for desirability: half of the items are positive statements about social relationships while negative statements form the other half. Items fall into four 10-item subscales: tangible support, appraisal support, self-esteem support, and belonging support.

## Results

### Comparison between Belgian and Italian Groups

Our first aim was to explore if the availability of legal marriage influences the ways in which LG couples manage their transitions to parenthood and their co-parental alliances (**Table 2**).

**Table 2:** Lausanne Trilogue Play (LTP) prenatal mean scores in the three groups (Gay men, Lesbian and Heterosexuals people) divided by country of residence.

		Gay couples		Lesbian couples		Opposite-sex couples	
				
Country	LTP scales	Mean	*SD*	Mean	*SD*	Mean	*SD*
**Belgium**	Co-parent playfulness	3.41	0.870	3.63	0.760	3.57	0.879
	Structure of the play	3.52	1.07	3.78	0.917	4.11	1.10
	Intuitive parenting behaviors	3.47	1.23	3.42	1.17	3.36	1.42
	Couple cooperation	3.47	0.624	3.63	0.683	3.71	0.897
	Family warmth	3.70	0.919	3.47	0.964	3.43	0.959
	Co-parental alliance	17.58	3.22	17.94	3.50	18.18	4.30

**Italy**	Co-parent playfulness	3.59	1.37	3.25	1.21	3.73	1.08
	Structure of the play	4.53	0.799	4.33	1.07	4.68	0.65
	Intuitive parenting behaviors	3.23	1.56	2.42	1.24	2.82	1.50
	Couple cooperation	4.00	1.00	4.00	0.852	3.68	1.13
	Family warmth	3.59	1.32	3.17	1.19	3.36	1.09
	Co-parental alliance	18.88	5.30	17.25	4.02	18.18	4.14


For the purpose of observing differences in the subgroups we have performed a MANOVA between the prenatal LTP scales scores of the three groups (Gay men, Lesbians and Heterosexuals) for each Country. Given the difference of age and duration of relationship between the two groups, we introduce the two variables into the model as a covariate. Results showed no difference related to “Sexual orientation” (Λ = 0.890; *F* = 1.265(5,51); *p* = 0.294) and a difference between the “Country” (Λ = 0.805; *F* = 2.473(5,51); *p* < 0.05) and “Duration of Relationship” (Λ = 0.750; *F* = 3.393(5,51); *p* < 0.05) also confirmed by the Bonferroni *Post hoc*; our first hypothesis has been confirmed.

The Between-Subjects Effects showed an effect of the “Country” variable on two LTP variables: “Structure of the Play” (*F* = 6.121; *p* = 0.016) and “Couple Cooperation” (*F* = 4.543; *p* = 0.038).

Looking at the means in the above table, Italians participants seem to observe the most suitable structure and timing of the play, and a more adequate couple cooperation.

In order to verify our second hypothesis, we performed an univariate two-ways ANOVA for the dyadic adjustment, social support and internalized homophobia (**Table 3**).

**Table 3:** Mean scores and standard deviations of Dyadic Adjustment and Social Support for each group and mean scores of Internalized Homophobia for the LG (Lesbian and Gay) participants divided in the two country groups.

		Gay men		Lesbians		Heterosexuals	
			
Country		Mean	*SD*	Mean	*SD*	Mean	*SD*
**Belgium**	DAS	105.3	11.6	112.3	14.9	112.3	13.9
	ISEL	89.3	11.3	89.2	13.2	92.2	16.2
	MISS-LG	1.8	0.56	1.3	0.49		

**Italy**	DAS	115.4	15.1	128.1	9.5	108.4	13.9
	ISEL	91.7	16.2	96.7	10.8	92.4	11.9
	MISS-LG	0.18	0.39	0.27	0.48		


About the dyadic adjustment, we discovered important differences by country of residence (*F* = 15.79; *p* = 0.000), sexual orientation (*F* = 11.43; *p* < 0.001) and their interaction (*F* = 11.01; *p* < 0.001). Bonferroni *Post hoc* showed a significant difference between Italian lesbian and heterosexual participants (*p* < 0.001) and Italian lesbians and gay men (*p* = 0.002). Regarding country of residence, Italian participants had more dyadic adjustments than Belgian. When running the same ANOVA for social support, no differences were revealed.

About the internalized homophobia, the two-ways ANOVA was only relevant for the country (*F* = 252.87; *p* < 0.001) and the interaction between country and sexual orientation (*F* = 9.72; *p* = 0.002). A deeper analysis showed that Italian lesbians and gay men had less internalized homophobia than Belgian ones (1.54 vs. 0.22 of average): in this context our hypothesis has not been confirmed.

### Correlation between Co-parenting Alliance, Dyadic Adjustment, Social Support and Internalized Homophobia in Homosexuals Participants (Lesbian and Gay Couples)

In order to observe the connection between the co-parental dimension, the couple adjustment, the social support and the degree of internalized homophobia among LG participants, we performed one Pearson’s correlation at a time (**Table 4**), per country of residence.

**Table 4:** Pearson correlations between co-parental alliance, dyadic adjustment, social support and internalized homophobia.

	Belgium			Italy		
		
	DAS	MISS-LG	ISEL	DAS	MISS-LG	ISEL
Co-parent playfulness	0.103	-0.176	0,340^∗∗^	0,411^∗∗^	-0,297^∗^	0,323^∗∗^
Structure of the play	0.103	0,316^∗∗^	0.13	0.107	-0.049	-0.050
Intuitive parenting behaviors	-0.155	0.027	-0.012	0,310^∗∗^	-0.216	0.208
Couple cooperation	0,214^∗^	0.185	0.120	0,435^∗∗^	-0,357^∗∗^	0,280^∗^
Family warmth	-0.140	0.027	0.192	0,360^∗∗^	-0,364^∗∗^	0,380^∗∗^
Co-parental alliance	0.003	0.104	0.160	0,409^∗∗^	-0,312^∗∗^	0,301^∗^


*^∗∗^*p* < 0.001, ^∗^*p* < 0.005.*

Analyses showed two different profiles. On one hand, we have the Belgian group for which the dyadic satisfaction significantly relates to couple cooperation: the couples who are involved in the “play of parenting” and share playfulness have higher level of social support.

On the other hand, the Italian group’s results showed that the Italian LG participants with better performances in co-parental alliance have higher score of dyadic satisfaction, and it seems to be connected with a lower level of internalized homophobia and a higher social support.

### Predictor of Co-parental Alliance in Lesbian and Gay Couples

So as to test the moderating role of dyadic satisfaction and the degree of internalized homophobia on the co-parental alliance, we used a hierarchical multiple regression analysis.

First, we included the dyadic satisfaction and internalized homophobia finding that both predict the co-parental alliance (*R*^2^ = 0.077, *p* = 0.022), as Dyadic Satisfaction and Dyadic Consensus show positive effect on the co-parental alliance (Dyadic Satisfaction β = 0.334, *p* < 0.001; Dyadic Consensus β = 0.309, *p* = 0.028). Then, we included interactions between variables and the influence of the country of residence: the model is still predictive and it explains 15% of the variance (*R*^2^ = 0.147, *p* = 0.003). This analysis displayed a specific effect on the Internalized Homophobia Identity scale linked to the country of residence (β = -0.341, *p* = 0.042).

## Discussion

This study purpose was to observe intuitive co-parental behaviors and relationship quality among LG couples who aim to become parents, compared with heterosexual peers. Our intent was to evaluate in which way the degree of internalized homophobia affects co-parental alliance, and if living in a country which has no legal system in favor of the LG community operates as a risk factor for the family-to-be.

Our sample cannot be defined as representative of the population because of its size and convenient nature. Most of Italian LG couples were members of LGBT associations, and probably more keen to show a positive image of lesbians and gay men. Nevertheless, it was complicated to reach couples out of this circuit without using specific Social Networks or accessing LGBT associations’ newsletters. Indeed, because of the absence of legal standards concerning the desire of parenthood for LG people, couples who want to become parents firstly contact these communities rather than “official” places (such as hospitals or social services) to get information. Listing results previously displayed, the first evidence is the difference of age and duration of relationship between Italian and Belgian participants. However, our statistical analyses with “age” as a covariate showed that the difference of age between the two groups did not explain the differences we observed between the groups. In regards of this absence of age effect, it is necessary to analyze this result in the light of the Italian socio-demographic context. As a matter of fact, according to an ISTAT report (?), Italy is characterized by a low and belated fertility, as the mean age of the first childbirth is over the age of 30. Thus, this difference between Belgian and Italian participants could illustrate the situation of couples who start to express their first desire for parenthood.

According to literature (Patterson, 2000; D’Amore et al., 2013), our preliminary analysis confirmed that there were no differences between lesbian, gay and heterosexual couples in terms of co-parental alliance. The only significant difference regards the Structure of Play, which evaluates the compliance to play by the game rules rather than expressing proper parental skills. The “Structure of Play” is also a variable that seems to be linked to the country of residence unlike the others LTP variables (Simonelli et al., 2012; D’Amore et al., 2013). The differences highlighted in our study appear to focus on the quality of the couple relationship, which varies from one group to another. Indeed, it seems that Italian lesbian couples show a higher level of dyadic adjustment than heterosexual and gay couples; this result confirms the data emerged from literature (Farr and Patterson, 2013). The degree of internalized homophobia of Italian participants is significantly lower than for the Belgian ones. A previous study (Lorenzi et al., 2015) already showed that Belgian lesbians and gay men express more internalized homophobia, despite Belgians live in a country with a more favorite legal environment for LG rights. Besides, the same study demonstrated that internalized homophobia had a strong relation with mental health disorders, such as anxiety and depression. Thus, it is an important variable to be evaluated, as it could be an indirect factor that could affect parental skills and then co-parental behaviors. In addition, from the beginning of the transition to parenthood, both of LG future parents have to face several stressful situations both inside their family (e.g., who the biological parent will be between them in case of artificial techniques), and out of it (e.g., the context in which they live in). They would not have the same custodial rights on their child, unlike heterosexual parents. Focusing on correlations, high levels of couple adjustment lead to better parental performances among both Italian and Belgian couples. At the same time, more social support helps couples to carry out the task with happiness rather than boredom. These positive influences correspond to Belsky’s (1984) theory about the multi-factorial vision of parenting. The incoherence among correlations by country is highlighted by the opposite influence that internalized homophobia has on parental alliance: in Belgium, better performances are characterized by higher levels of internalized homophobia, even if it is a risk factor for several negative issues. It seems that Belgian LG couples with high internalized homophobia have more cooperative parental behaviors and better adjustment to the game. It is possible to claim, without generalizing (because of the sample size), that Belgian LG couples with a high level of internalized homophobia invest themselves in their own relationship and they strictly comply with the LTP roles and rules to offer the best performance. On the contrary, Italian performances seem to confirm positive patterns between a low level of internalized homophobia and parental behaviors. These discordances between Belgian and Italian participants may also be explained by their difference of age. Indeed, as Belgian participants were younger, we can also assume that they were less mature and more stereotypical than Italian participants. Further research should evaluate if the link between internalized homophobia and parental alliance persists when Belgians get older and when couples become actual parents.

Besides, this study adds information about the negative influence of the internalized homophobia on co-parental alliance. Even if they must be carefully read into, these results are of great interest: they suggest that sexual stigma has an impact on the capability of managing co-parenting. Indeed, internalized homophobia is a mandatory step that every gay and lesbian person has to face across the construction of his/her own identity (Montano, 2007). The identity component denotes the attitude to express a negative consideration of oneself as gay or lesbian, and it is important to assess how much this passage is overrun during the evaluation of parental skills among LG couples.

This study showed that the civil rights for Belgian LGBT people seem not to be enough to ensure their well-being; homophobia and sexual stigma are still often expressed in an implicit, subtle and indirect way. Our data offer some valuable indications about implications of internalized homophobia in the process of the identity construction and of the impact on the inter-generational transmission of psychopathology. As a matter of fact, the identity dimension of the MISS-LG, which “*corresponds to an enduring propensity to have a negative self-attitude as homosexual and to consider sexual stigma as a part of a value system and identity*” (Lingiardi et al., 2012, p. 1196), correlates with lower levels of co-parental alliance. This suggests that an individual, with a negative self-esteem due to her/his homosexuality, may find it difficult to manage the role of parent, because he/she always considers that role through a heteronormative perspective.

These themes need a further and closer examination, in particular in these early periods of lifecycle and with a strong collaboration among the countries.

## Ethics Statement

Ethics committee of “Faculté de Psychologie, Logopédie et Sciences de l’Education” University of Liege. Project accepted the 10/31/2013. Participants sign a consent about the research protocol explaining the project details as well as the relative finalities, nature of data required, information’s about participants rights.

## Author Contributions

MM: Study conception and design, data collection and analysis, interpretation of results and write up. AB: Data analysis, interpretation of results and write up. PP: Data collection, interpretation of results and write up. AS: Study conception and design, data analysis, interpretation of results and write up.

## Conflict of Interest Statement

The authors declare that the research was conducted in the absence of any commercial or financial relationships that could be construed as a potential conflict of interest.

## Footnotes

1 This research has obtained the approval of the Ethics Committee of the University of Liège (Project accepted the 10/31/2013).

2 Service de Psychologie clinique de l’enfant et de l’adolescent, University of Liège and Department of Developmental and Social Psychology, University of Padua.

3 Sexuality: The sexuality dimension describes the pessimistic evaluation of intimate gay or lesbian relationships’ quality and duration and a negative conception of gay or lesbian sexual behaviors (e.g., “I don’t believe in love between homosexuals”; “When I have sex with a woman, I feel awkward”). Identity: The identity dimension corresponds to an enduring propensity to have a negative self-attitude as homosexual and to consider sexual stigma as a part of a value system and identity (self-stigma; e.g., “I’d prefer to be heterosexual”; “If it were possible, I’d do anything to change my sexual orientation”). Social Discomfort: Social discomfort is the fear of public identification as a lesbian or gay man in the social context, disclosure in private and professional life, and negative internalized beliefs regarding religious, moral, and political acceptability of homosexuality (e.g., “At University (and/or at work), I pretend to be heterosexual”; “It’s difficult for me to say that I’m lesbian/gay, even to someone I know”).
